# A 3D-Printed Microfluidic Device for qPCR Detection of Macrolide-Resistant Mutations of *Mycoplasma pneumoniae*

**DOI:** 10.3390/bios11110427

**Published:** 2021-10-29

**Authors:** Anyan Wang, Zhenhua Wu, Yuhang Huang, Hongbo Zhou, Lei Wu, Chunping Jia, Qiang Chen, Jianlong Zhao

**Affiliations:** 1 College of Metrology and Measurement Engineering, China Jiliang University, Hangzhou 310018, China; p1902085248@cjlu.edu.cn; 2 State Key Laboratory of Transducer Technology, Shanghai Institute of Microsystem and Information Technology, Chinese Academy of Sciences, Shanghai 200050, China; wuzhx@mail.sim.ac.cn (Z.W.); 1000479741@smail.shnu.edu.cn (Y.H.); zhouhb@mail.sim.ac.cn (H.Z.); jlzhao@mail.sim.ac.cn (J.Z.); 3 College of Life Sciences, Shanghai Normal University, Shanghai 200233, China

**Keywords:** *Mycoplasma pneumoniae*, macrolides, resistance mutations, microfluidic, 3D-printed, sample-to-answer, qPCR

## Abstract

Mycoplasma pneumonia (MP) is a common respiratory infection generally treated with macrolides, but resistance mutations against macrolides are often detected in *mycoplasma pneumoniae* in China. Rapid and accurate identification of *mycoplasma pneumoniae* and its mutant type is necessary for precise medication. This paper presents a 3D-printed microfluidic device to achieve this. By 3D printing, the stereoscopic structures such as microvalves, reservoirs, drainage tubes, and connectors were fabricated in one step. The device integrated commercial polymerase chain reaction (PCR) tubes as PCR chambers. The detection was a sample-to-answer procedure. First, the sample, a PCR mix, and mineral oil were respectively added to the reservoirs on the device. Next, the device automatically mixed the sample with the PCR mix and evenly dispensed the mixed solution and mineral oil into the PCR chambers, which were preloaded with the specified primers and probes. Subsequently, quantitative real-time PCR (qPCR) was carried out with the homemade instrument. Within 80 min, *mycoplasma pneumoniae* and its mutation type in the clinical samples were determined, which was verified by DNA sequencing. The easy-to-make and easy-to-use device provides a rapid and integrated detection approach for pathogens and antibiotic resistance mutations, which is urgently needed on the infection scene and in hospital emergency departments.

## 1. Introduction

Respiratory infection is the most common type of infection in the clinic and the risk of illness is consistent year-round. The pathogens causing pneumonia are often *mycoplasma pneumoniae* [1], especially in children and adolescents [2,3]. Mycoplasma pneumonia (MP) has a longer disease duration and more severe symptoms of the lung compared with other pneumonia. Macrolide is the preferred drug to treat MP [4], but macrolide resistance is becoming a widespread problem because of antibiotic abuses. According to the recent reports, the detection rate of the drug-resistant gene of *mycoplasma pneumoniae* is over 90% in China [5,6]. The primary mechanism leading to macrolide resistance to *mycoplasma pneumoniae* is the point mutation at sites 2063 and 2064 in domain V of 23S ribosomal RNA, especially the mutation of A2063G [7]. The difference of mutant types and sites can cause different macrolides resistance [8]: both A2063G and A2064G mutant strains are highly resistant to erythromycin and azithromycin; the A2064G mutant strain is more resistant to thromycin than A2063G; the A2063T mutant strain is also highly resistant to erythromycin, but has a low resistance to azithromycin and thyromycin. Therefore, it is pivotal to rapidly detect *mycoplasma pneumoniae* and its drug-resistant sites, which can help to diagnose respiratory infection and provide precise guidance on treatment and the use of medication.

Traditionally, the golden standard of microbial pathogen identification is to culture human sputum, blood or other samples collected from the human body with specific media and afterwards judge the type of pathogen by its appearance and morphology with experience [9]. The method is time-consuming, and it is easy to cause false-negative results because of missed detections. The emerging microbial pathogen detection technology can overcome the shortcomings of the culture method, which is divided into three categories: molecular diagnostic technology [10], immunoassay [11] and biosensors technology [12,13]. The detecting targets of molecular diagnostic technology are nucleic acids, so it can directly reveal the genetic information of the pathogen. The detection can be implemented by hybridization [14], polymerase chain reaction (PCR) [10], isothermal amplification [15,16], CRISPR [17] or high-throughput sequencing [18]. Among these methods, PCR is the most widely used, especially quantitative real-time PCR (qPCR), because it provides fast, quantitative, specific and sensitive detection, which can precisely distinguish a single-base mutation in the gene sequence. However, qPCR has a cumbersome multi-step preparation before nucleic acid amplification. And there are three challenges during the operations, including personnel operating deviations, sample contaminations and the risk of operator infections. With the development of microfluidics technology, these problems can be solved by building an integrated automation platform.

Recently, several microfluidic approaches have been developed for the identification of pathogen and antibiotic resistant mutations, such as single-cell trap, culture and imaging [19], identifying and detecting the pathogen markers in droplets by DNAzyme-based sensors [20], aptamer-Ag10NPs detection with bright field imaging [21] and nucleic acid samples preparation and amplification [10,22,23,24]. The sample-to-answer systems are also available on the market, such as the BioFire FilmArray and Cepheid GeneXpert, which are based on rapid, specific and sensitive PCR assays [10]. In order to further increase efficiency and reduce costs, newer microfluidic devices were developed to perform the complete procedure of the nucleic acid assay, including cell lysis, DNA purification, gene amplification and amplicon detection [10]. One category consists of centrifugal microfluidic devices [22], the other includes devices with micro-pumps, micro-valves, and reaction chambers [23,25]. The accelerated development in the field needs rapid and cheap fabrication approaches for a proof of concept.

3D printing is a promising technology to fabricate microfluidic device because of its many advantages, including the ability to create stereoscopic architectures directly and rapidly, as well as the cheap, quick implementation of the design and the optimization. Currently, microfluidic 3D printing for biological assays and clinical tests is still in a start-up stag, the resolution of 3D printing, the biocompatibility of resin and multi-materials integration still need investigation [26,27].

In this study, a novel 3D-printed device used for qPCR detection of macrolide-resistant genes of *mycoplasma pneumoniae* was proposed. The device automatically mixed the sample with the reagent and then the mixture was evenly dispensed into multiple PCR chambers in which the specified primers and probes had been preloaded. Afterwards, the device was transferred to the homemade qPCR system. Two kinds of single-base mutations of *mycoplasma pneumonia* were identified with high speed, specificity and convenience.

## 2. Materials and Methods

### 2.1. Device Design and Fabrication

**Figure 1:**
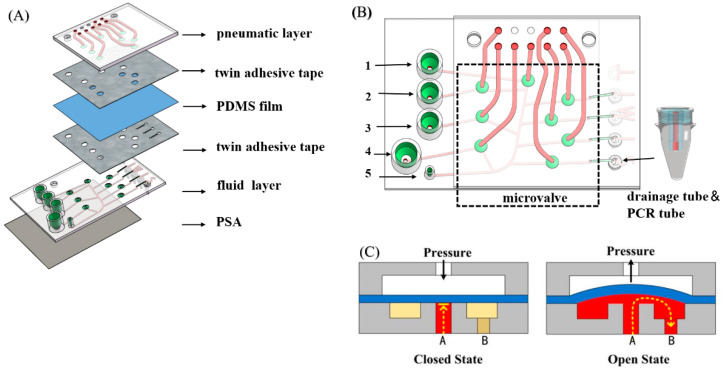
(**A**) Exploded diagram of the device. (**B**) Schematic illustration of the device. One to three are reagent reservoirs, 4 is a vent and 5 is a Luer taper connected to a syringe pump. Polypropylene PCR tubes can be fitted with the connectors on the device. (**C**) Cross section of a microvalve and its working principle.

The device diagram is shown in Figure 1A, which consists of 6 layers. The pneumatic layer and the fluid layer were fabricated by stereolithographic (SLA)3D printing (UnionTech Lite800, UnionTech Inc., Shanghai, China). A 200-μm-thick PDMS membrane was sandwiched between the pneumatic layer and the fluid layer. Two pieces of twin adhesive tapes (ARseal™ 90880, Adhesives Research Inc., Glen Rock, PA, USA) were patterned using laser etching. During assembly, two sides of the tape were adhered respectively to the 3D-printed layer and the PDMS membrane, both of which were treated with plasma in advance. After the pneumatic layer, the PDMS membrane and the fluid layer were assembled; a pressure sensitive adhesive tape (PSA, 3M 9795R) was pressed tightly onto the bottom of the fluid layer to close the flow channels.

The assembled device is shown in Figure 1B, with a length of 100 mm and a width of 75 mm. Five is a simple Luer taper, used to connect the flow channel to a syringe pump. One to three are sample reservoirs, which are respectively filled with mineral oil, the PCR mix, and a mixed solution containing sample. Four is a vent for balancing the internal and the external pressure of the device. There are eight microvalves in the device. Functions including mixing, dispensing and oil adding can be performed by manipulating the syringe pump and these microvalves. The cross-sectional structure of the microvalve is shown in Figure 1C. The upper pneumatic layer and the lower fluid layer are separated by a PDMS membrane, whose deformation direction determines the opening and closing of the microvalve. When a positive pressure is applied to the upper channel, the bend-down PDMS membrane blocks the fluid flow from the A channel. When a negative pressure is applied, the PDMS membrane bends up so the fluid can flow from A to B. On the right lower side of the device there are five drainage tubes and connectors which can fit PCR tubes tightly. Ventholes designed at the edge of the base of drainage tubes ensure that the solution can flow smoothly into PCR tubes.

### 2.2. System Setup and Workflow

**Figure 2:**
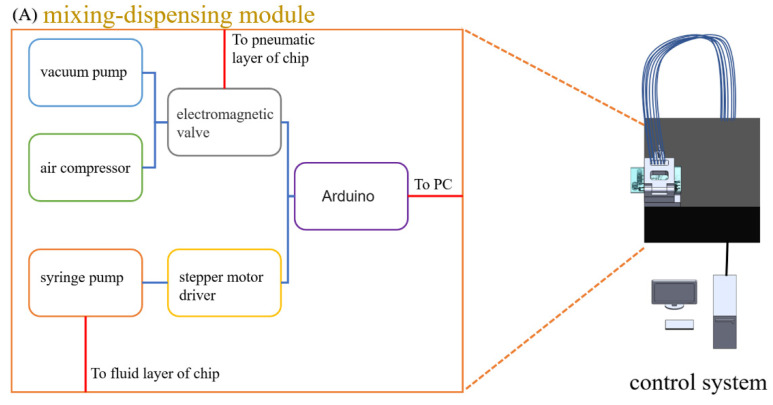

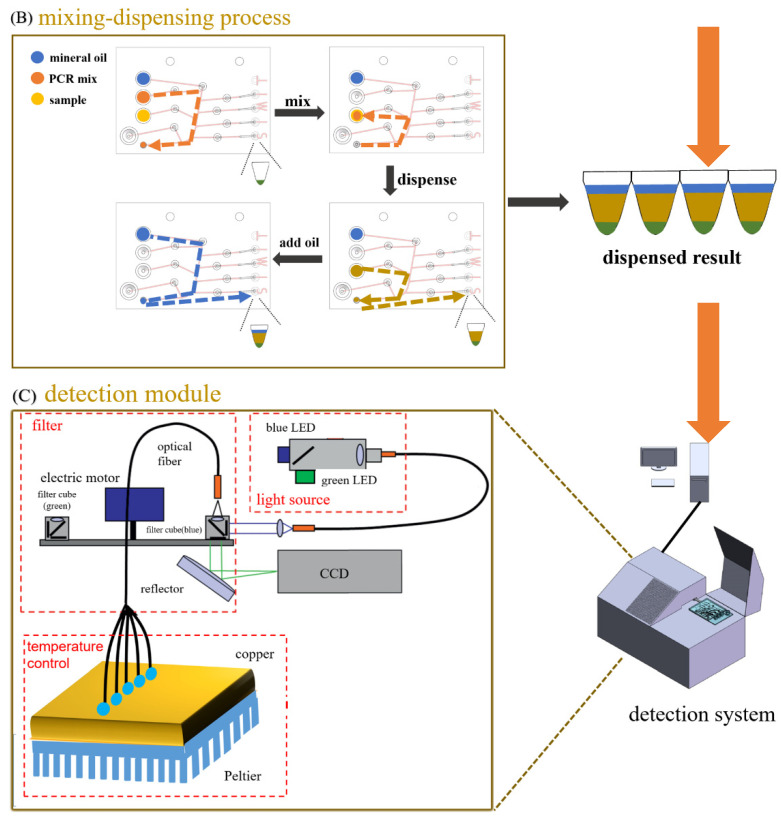
(**A**) Structure diagram of the mixing-dispensing module. (**B**) Schematic illustration of mixing and dispensing process. (**C**) Structure diagram of the detection module.

In order to achieve the goal of sample-to-answer, a set of systems were developed for the automation of both the sample preparation and qPCR, which contained a mixing-dispensing module and a detection module. As shown in Figure 2A, the mixing-dispensing module was composed of a syringe pump, an air compressor, a vacuum pump and a control circuit. The syringe pump was connected to the fluid layer of the device to control the advance and retreat of the fluid, and the vacuum pump and the air compressor were connected to the pneumatic layer of the device to control the opening and closing of the microvalve. As shown in Figure 2C, the detection module was composed of a LED light source, a filter unit, a photomultiplier tube (PMT) detector and a temperature control unit. The spectral coverage of the light source was from the blue region to the green region. The beam from the light source was delivered with an optical fiber and then was expanded by a lens. Next, the expanded beam went through the filter cube and was delivered with an optical fiber to illuminate the PCR solutions in tubes. Afterwards, the excited fluorescent beam was delivered back into the filter tube and went through the dichroic mirror and the barrier filter of the tube and was finally detected by PMT. The PCR tubes on the device could be inserted into the metal slots on the copper base of the temperature control unit, where a Peltier module was used for precise temperature control (Figure S1). During each temperature cycle, the fluorescent signals of five PCR tubes were recorded sequentially with the PMT detector.

The workflow was divided into two steps. The first step was mixing the sample with PCR mix and then dispensing the mixtures into four tubes. The PCR mix was sucked into the tube connected with the syringe from reservoir 2 and then infused into reservoir 3 to mix with the sample. After the mixing was completed, a certain amount of the mixed solution was sucked by the syringe pump and dispensed to the 4 PCR tubes in sequence. The PCR tubes were preloaded with primers and fluorescent probes. In order to balance the air pressure inside and outside the device after each dispensing, the valve of the vent would be opened. The mineral oil was finally added to each PCR tube to prevent the aerosol contamination during PCR thermal cycling. At the second step, the device was transferred to the detection module, in which qPCR detection was performed. The curves of both the fluorescence intensity and temperature of the PCR tubes were displayed and recorded in real time with the homemade software.

### 2.3. Analyzing the Effects of Materials and Coatings on PCR

The photosensitive resins used in the device fabrication were Somos WaterClear Ultra 10122 and Somos WaterShed XC 11122. After 3D printing, some devices were treated with varnish to incease the transparancy. In order to analyze the effects of the resins and the coatings on the PCR, a piece of 2 × 2 × 3 mm^3^ was cut from the 3D-printed devices, which were made with different resins, coated or uncoated, and was immersed in 30 μL PCR reagent for 12 min. Also, a piece of 2 × 2 mm^2^ PSA was immersed in 30 μL PCR reagent for 12 min. Next, the original and treated PCR reagents were put in LightCycler^®^ 480 (Roche Diagnostics, Rotkreuz, Switzerland) to carry out qPCR analysis. In order to further investigate the effects of photosensitive resins on PCR, the materials were kept in the PCR reagents to carry out qPCR analysis. In all assays, the PCR regents mixed and dispensed by hand were used as the control.

### 2.4. qPCR Detection of Macrolide-Resistant Mutations of Mycoplasma pneumoniae

In the early stage of proof of concept, the gene sequences of *mycoplasma pneumoniae* with the mutation of A2063G in 23S rRNA was inserted into a pUC57 vector and cloned. The cloned plasmids were used as the substitute of the clinic samples. The plasmid sequence was described in the supplementary material. Finally, three clinical samples of throat swabs were analyzed, which were kindly provided by Prof. Min Li’s group from Renji Hospital affiliated to Shanghai Jiao Tong University.

The primers and the probes were respectively designed according to the conserved sequence of the P1 gene, the mutation sequence of A2063G and A2064G in 23S rRNA. The TaqMan MGB probes were labeled with FAM. In the qPCR assay with the microfluidic device, 4 PCR tubes were preloaded with the different primers and probes and 1 tube, as the negative control, was preloaded with all PCR reagents except the sample. The final composition of the PCR solution after the reagents’ preparation is depicted in Table 1.

In the assay, the 103.5 μL of sample, 135 μL of the PCR mix and 300 μL of mineral oil were added into the different reagent reservoirs of the device. After the sample preparation was completed, the device was transferred to the detection module for qPCR. The thermocycling protocol included an initial denaturation at 95 °C for 2 min, followed by denaturation at 95 °C for 30 s, annealing at 55 °C for 40 s andan extension at 72 °C for 10 s, repeated for 45 cycles. 

### 2.5. Sensitivity of Detection System 

In order to test the detection sensitivity of the system, A2063G plasmid was used as a template for ten times dilution to obtain the reaction premixes containing the plasmids at different concentrations. In different premixes, the sample contents were 10,000 copies, 1000 copies, 100 copies and 30 copies, respectively. Then the PCR reaction solution was prepared with the above diluted samples and amplified in the homemade qPCR instrument.

## 3. Results

### 3.1. Characterization of Bonding Strength and Microvalve Performance

In order to integrate pneumatic microvalves in the device, it is necessary to assemble a pneumatic layer, a PDMS membrane and a fluid layer together. In the traditional manufacturing method of PDMS chip [28], multi-layer PDMS can bond together after the plasma treatment, which can withstand the pressures up to 300 kPa without delamination [29]. However, the cured photosensitive resin cannot be easily bonded this way. Therefore, adhesive tapes were used to assemble the device. To avoid liquid leakage, the bonding strength between different layers was tested. In the fluid layer, PSA bore the pressure change caused by the movement of the syringe piston. In the pneumatic layer, the twin adhesive tape and PDMS bore the positive pressure applied when closing the microvalve. 

**Figure 3:**
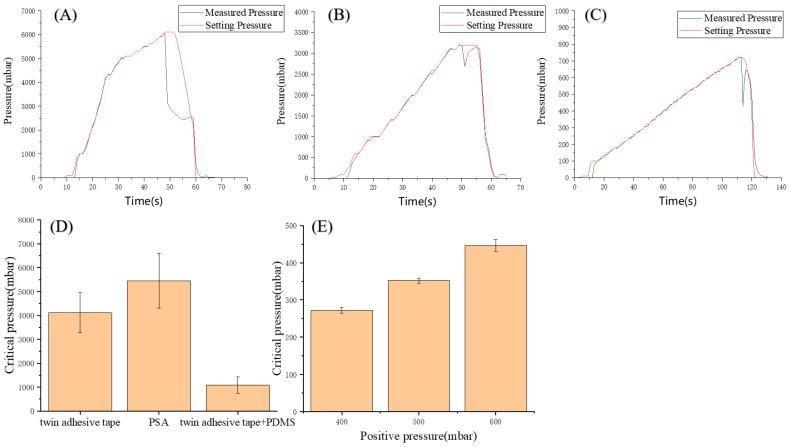
Typical bonding test curves of PSA (**A**), twin adhesive tape (**B**) and PDMS (**C**) with the chip. (**D**) The critical pressure that the different bonding can withstand. (**E**) The critical pressure that the microvalve can withstand at a given positive pressure. (n = 3).

Pressure test chips with only inlet but no outlet were fabricated (Figure S2). The inlet radius was 1 mm and the straight channel was 18 mm long and 0.8 mm wide. The total area of channel was 17.54 mm^2^. Tapes were cut into 5 mm wide strips and bonded with the back of the chip to close the channel. The inlet was connected to the precision air pressure control system (MFCS^TM^-EZ, Fluigent). Figure 3A–C shows that when the pressure reached the critical pressure, the measured pressure dropped off cliff-like, which means that the tapes separated from the test chip. All cases were tested three times, and the critical pressure was recorded for each (Figure 3D). As shown in Figure 3D, the minimum critical pressure of PSA is 4133mbar. The minimum force that the PSA can withstand was calculated to be 7.2N. According to the equation of the ideal gas state, pV = nRT, where p is the pressure, V is the volume, n is the amount of substance, R is the gas constant and T is the absolute temperature, the product of the pressure and the volume of a certain amount of gas is constant when the temperature is constant. The total volume of the syringe and the connecting pipe is 16.5 cm^3^, ignoring the volume of the chip channel. When the mixing-dispensing module was working, the maximum distance that the syringe pump pushed the piston was 8mm and the volume was reduced by 1.23 cm^3^, assuming that eight microvalves were closed at the same time. The pressure was increased by about 80 mbar. The channel area from the Luer taper to the eight microvalves is 137.6 mm^2^, so the PSA bore force is 1.1N, much less than 7.2 N. This showed that the PSA could ensure the chip would not be layered or leak during the working process. In the experiment, no liquid leakage was observed.

The microvalve was a key structure to ensure that the samples can be mixed and dispensed correctly, so its performance was evaluated. According to the test results in Figure 3D, the minimum pressure that the PDMS can withstand was 723 mbar. Therefore, the calculated minimum force that the PDMS can withstand was 1.3 N. Because the area of the microvalve is 19.6 mm^2^, the positive pressure applied to the microvalve cannot exceed 647 mbar. Since the pressure that the twin adhesive tape can withstand was much greater than the PDMS, we did not need to calculate. Based on the calculated results, 400 mbar, 500 mbar and 600 mbar were respectively chosen as the positive pressure to test the performance of the microvalve. As shown in Figure 3E, according to the difference of the positive pressure, the pressure that the microvalve can withstand was 272 mbar, 352 mbar and 446 mbar, respectively. Theoretically, the pressure that the microvalve can withstand should be equal to the applied positive pressure. However, the surface of the 3D-rinted products was very rough (Figure S3). This caused a decrease in the performance of the microvalve. The Quake valve based on the 3D printing also encountered the same problem, and the non-smooth wall surface would affect the closing performance of the Quake valve [30]. However, due to the advantages of the microvalve with this structure, the actual performance was about 70% of the theoretical value. Therefore, it is very worthwhile to sacrifice the closing performance to reduce the difficulty of microvalve construction. The positive pressure in the experiment was usually 500 mbar, which can withstand pressure of 352 mbar far greater than 80 mbar, so the microvalves can ensure the normal progress of the mixing and dispensing.

### 3.2. Effects of Materials and Coatings on PCR

**Figure 4:**
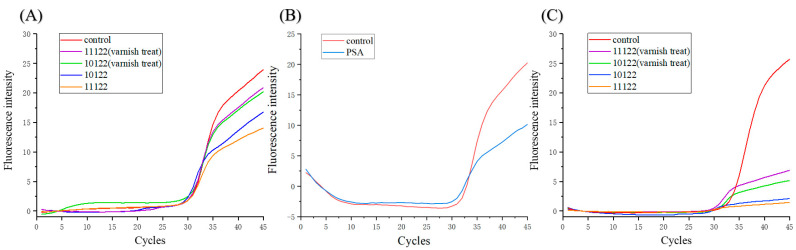
The typical qPCR curves after the PCR reagent contacted photosensitive resins (**A**) and PSA (**B**) for 12 min. (**C**) The qPCR curves of the PCR reagent in which photosensitive resins were immersed for PCR thermocycling.

So far, researchers have developed many photosensitive resins used for 3D printing, but whether these resins can be used as the container for biochemical reactions has not been tested. In addition, in order to enhance the transparency of the photosensitive resin, the cured product will be sprayed with the varnish. The effect of the coating on the biochemical reactions is unknown. It has been found that photoinitiator in photosensitive resins can inhibit the PCR reaction [31], so ultraviolet light was irradiated before the experiment to remove the remaining photoinitiator. As shown in Figure 4A,B, the photosensitive resin and the PSA had a certain inhibitory effect on the PCR reaction. However, the Ct value of each curve was nearly same, indicating that the plasmid was hardly adsorbed by the channels of the device. From Figure 4C, it can be seen that the final fluorescence intensity decreased significantly compared with the control, which might be because the resin immered in the PCR agent affected the activity of the polymerase. The heating might make small molecules in the photosensitive resin escape and inactivate the enzyme. The photosensitive resin sprayed with varnish could slightly increase the final fluorescence intensity. It was spectuated that the varnish coating of the device made the internal surface of channels more smooth and reduced the enzyme adsorption and small molecule escape. However, the improvement of the varnish coating was not obvious. In order to reduce the inhibitory effect of photosensitive resin on the PCR reaction, the reagent was prepared in the 3D-printed device and was then dispensed into the polypropylene PCR tubes to avoid contact between the reagent and the photosensitive resin during PCR amplification.

### 3.3. Sample Preparation

**Figure 5:**
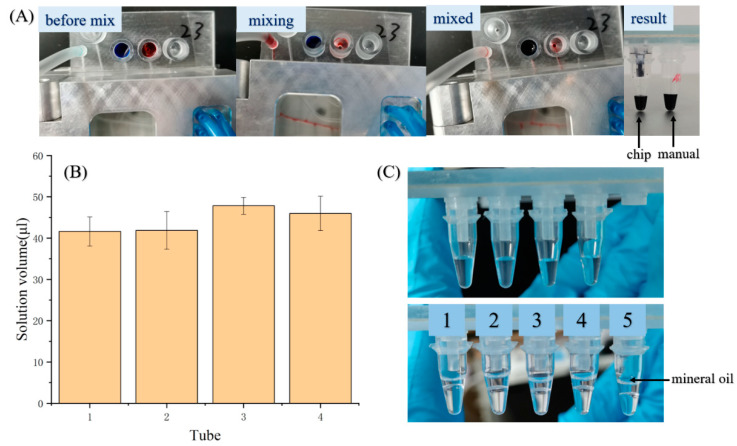
(**A**) Snapshots of dispensing process. (**B**) The reagent volume in PCR tubes after the preparation with the device. n = 3. (**C**) Photograph of the prepared reagents in the PCR tubes.

To complete qPCR sensitively and correctly, it is crucial that the sample and the PCR mix are mixed well and dispensing evenly. The mixing performance was evaluated. First, the red dye and blue dye were put in the reagent reservoirs, respectively, and then the dye solutions were mixed automatically by pushing and pulling the piston of the syringe pump with the program. The mixing result is shown in Figure 5A. The mixed solutions produced with the device and by hand are shown in the left and in the right, respectively. It can be clearly seen that both mix effects are similar, so the mixing with the device can meet the requirements. The dispensing of the solution mainly depends on the synergy between the microvalve control and the syringe pump, so the operation steps and the parameters were optimized in the preliminary experiments and the automatic process program was determined. To evaluate the dispensing performance, two solutions of 110 μL each were added into the two reservoirs and the volumes of the solution dispensed into PCR tubes were analyzed. As shown in Figure 5B, the amount of dispensed solution had good uniformity. Figure 5C shows the photographs of the dispensing results before and after adding oil, where tube five was for the manual oil added, and the rest of four tubes were for the oil added with the device.

### 3.4. Macrolide-Resistant Mutations Detection

**Figure 6:**
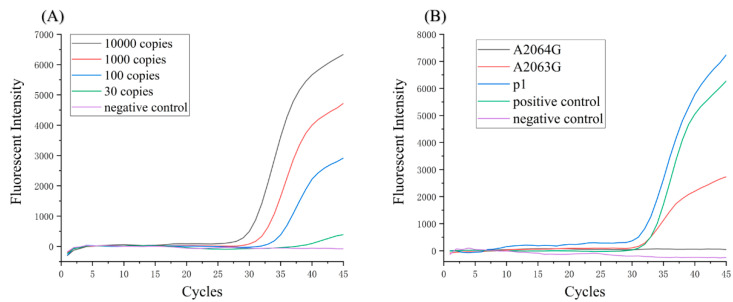
(**A**) Detection sensitivity of the homemade detection system. (**B**) The typical qPCR curves of the plasmid sample.

The sensitivity test result of the homemade qPCR instrument is shown in Figure 6A. The curve of 100 copies/reaction is an obvious S-shaped curve, which proves that the sensitivity of the detection system reaches 100 CFU/reaction. 

The detection of macrolide-resistant mutations of *Mycoplasma pneumoniae* took 80 min in total, 12 min for preparation and 68 min for qPCR. Three tests were conducted and the results were all positive for A2063G (Figure S4). The typical qPCR curves are shown in Figure 6B. A2063G and the negative control showed negative curves. P1, A2063G resistance mutation and positive control showed obvious positive curves, whose Ct values were 30.8, 30.7 and 30.1, respectively. According to the detection criteria (see the supplementary material), the analyte was *Mycoplasma pneumoniae* and the type of mutation was A2063G, which were consistent with the A2063G plasmid sample.

### 3.5. Detection of the Clinical Samples

**Figure 7:**
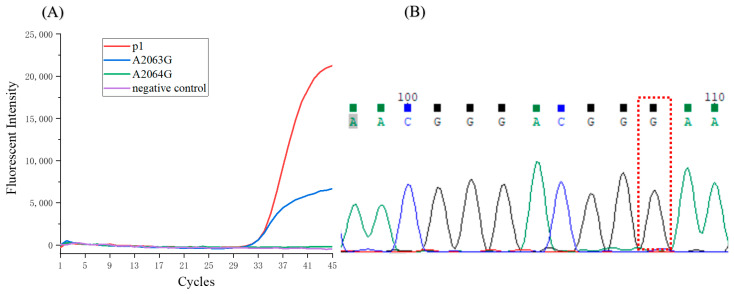
(**A**)The qPCR curves of the clinical sample. (**B**) The sequencing result of the clinical sample.

The detection based on the device demonstrated high sensitivity and accuracy in the plasmid assays. Subsequently, three clinical samples of throat swabs were analyzed with the device. The qPCR curves of one clinical sample are shown in Figure 7A. A2063G mutations of *mycoplasma pneumoniae* were determined. In order to verify the result, the amplified product was analyzed using DNA sequencing. The result also indicated the A2063G mutation (Figure 7B). Two more clinical samples were analyzed, both using the approach based on the device and DNA sequencing, and consistent results were obtained (Figure S5). This approach, based on a 3D-printed device, can accurately detect the macrolide-resistant mutation of *mycoplasma pneumoniae*.

## 4. Discussion

Nowadays, *Mycoplasma pneumoniae* has a variety of macrolide-resistant mutations, and the mutations of A2063G and A2064G are the most common types. The detection of macrolide-resistant mutations using qPCR is very important for clinical treatment. However, traditional qPCR detection requires manual sample preparation and special PCR rooms. Also, it may cause the risks of sample contamination and operator infection. Therefore, it is a good choice to use an integrated microfluidic device instead of manual operations.

The 3D printed microfluidic device combined with the mixing-dispensing module and detection module is proposed to easily realize sample-to-answer detection. Using the modules, the device can mix reagents and dispense the mixture to different PCR tubes and achieve multi-channel qPCR detection. In this way, *Mycoplasma pneumoniae* and its mutation type can be identified. 

Differing from the previous works, the PCR chambers were not constructed inside the device. Instead, the connectors at the end of channels were designed and fabricated. The commercially available PCR tubes could be fitted tightly with the connectors and performed as PCR chambers, which effectively reduced the inhibitory effect of the device materials on the PCR. Moreover, the mutually independent PCR chambers avoided the contamination problem, making the detection results more credible. However, limited by the size of the PCR tubes, the size of the device cannot be reduced, which is a problem to be addressed in the future. 

The microvalve was an important part of the mixing-dispensing module. The proposed microvalve structure can be fabricated with one step using 3D printing. The PDMS membrane was sandwiched between the pneumatic layer and the fluid layer to construct the microvalve structure. The microvalve was a normally closed valve and the positive pressure applied to the pneumatic layer further ensured that the membrane fully covered the inlet of the microvalve and completely blocked the flow of liquid. Compared with the Quake valve, which is a normally open valve, less positive pressure is needed to close the valve.

In addition, with the cooperation of microvalves, only one syringe pump was needed to drive several fluids, which greatly reduced the number of syringe pumps used. At present, the dead volume of the valves cannot be eliminated and the fluid in the annular chamber of the valve cannot be discharged completely, which results in the loss of part of the solution during each dispensing. Due to the uncontrollable amount of lost solution, there are slight differences in the amount of dispensed solution, which in turn affects the uniformity of the dispensing result. More efforts should be made to improve the performance of the microvalve.

## 5. Conclusions

In summary, a sample-to-answer device and its periphery were developed to detect macrolide-resistant mutations of *Mycoplasma pneumoniae*, including A2063G and A2064G. Sample preparation, including mixing and dispensing, were achieved with the device on the mixed-dispensing module and, afterwards, the device was transferred to the fluorescence detection module to carry on qPCR. In the assays both of the plasmid and the clinical sample, the approach based on the device showed high sensitivity and accuracy. 

In fabrication, the 3D printing technology provides conveniences such as rapid, easy-to-make and cheap production. Therefore, the design can be easily changed according to the requirements. The device has several similar structure units, so the detection of more channels is easy to achieve by increasing the numbers of structure units.

The device integrated different materials, taking advantages of their benefits. The photosensitive resin easily processes stereoscopic structures. The elastic PDMS membrane is used for the microvalve structure. The PCR tubes reduced the inhibitory effects of the material on the PCR. 

## Figures and Tables

**Table 1:** qPCR solution composition after the preparation with the device (besides PCR mix).

	Primer	Probe	Other	Sample Treated by the Device
Tube 1 (conserved sequence)	P1 gene	P1 gene	/	Plasmid/clinical sample
Tube 2 (A2063G mutation)	23S rRNA	A2063G	/	plasmid/clinical sample
Tube 3 (A2064G mutation)	23S rRNA	A2064G	/	plasmid/clinical sample
Tube 4 (positive control)	23S rRNA	A2063G	A2063G plasmid	plasmid/clinical sample
Tube 5 (negative control)	23S rRNA	A2063G	water	/

## Data Availability

The data presented in this study are available upon request from the corresponding author.

## Supplementary Materials

The following are available online at https://www.mdpi.com/article/10.3390/bios11110427/s1. Figure S1. (A)Temperature curve of the homemade instrument. The white line is the temperature condition of fluorescence detection. Whenever the temperature curve drops below the white line, the fluorescence intensity is measured once; Figure S2. (A) The 3D model of the test chip. scale bar is 10mm. Closed channel by PSA(B), twin adhesive tape(C) and PDMS&hollowed twin adhesive tape(D); Figure S3. Snapshot of microvalve. scale bar is 500μm; Figure S4. The qPCR curves of other two plasmids samples; Figure S5. The qPCR curves and sequencing results of other two clinical samples(No.129(A) and No.112(B)).
