# Supplementary material for: A 3D-Printed Microfluidic Device for qPCR Detection of Macrolide-Resistant Mutations of Mycoplasma pneumoniae
Source: Biosensors (Basel). 2021 Oct 29;11(11):427. doi: 10.3390/bios11110427 (PMC8615801; doi:10.3390/bios11110427)
Supplement: Supplementary file 1 [file biosensors-11-00427-s001.zip › biosensors-1402696-supplementary.pdf]

biosensors

MDPI

Supplementary Materials

1. The plasmid sequences of mycoplasma pneumoniae with the mutation of A2063G in23S rRNA:

GATGTATATGGGGTGACACCTGCCCAGTGCTGGAAGGTTAAAGAAGGAGGTTAGCGCAAGCGAAGCTTTTAACTGAAGCCCCAGTGAAC
GGCGGCCGTAACTATAACGGTCCTAAGGTAGCGAAATTCCTAGTCGGGTAAATTCCGTCCCGCTTGAATGGTGTAACCATCTCTTGACTGT
CTCGGCTATAGACTCGGTGAAATCCAGGTACGGGTGAAGACACCCGTTAGGCGCAACGGGACGGG(A2063G)AAGACCCCGTGAAGCTTT
ACTGTAGCTTAATATTGATCAGGACATTATCATGTAGAGAATAGGTAGGAGCAATCGATGCAAGTTCGCTAGGACTTGTTGATGCGAAAGG
TGGAATACTACCCTTGGTTGTGTGCTGTTCTAATTGGTAACTGTTATCCAGTTTCAAGACAGTGTTAGGTGGGCAGTTTGACTGGGGCGGT
CGCCTCCTAAAAGGTAATGGAGGCGTACAAAGGTACCTTCAGTACGGTTGGAAATCG

2. Detection criteria of macrolide resistant mutation of mycoplasma pneumoniae:

FAM fluorescent labeling was used：

Reaction 1: positive control, FAM channel had Ct value and Ct≤43;

Reaction 5: negative control, FAM channel had no Ct value or Ct＞43;

When the above results are satisfied in one experiment, the detection results are effective. Otherwise, this experiment is invalid.

When the detection results are effective, analyze the results:

Reaction 2: for P1, If the typical S-type amplification curve is obtained and Ct≤43, it is positive for Mycoplasma pneumoniae;

Reaction 3: for A2063G, If the typical S-type amplification curve is obtained and Ct≤43, it is positive for A2063G mutation;

Reaction 4: for A2064G, If the typical S-type amplification curve is obtained and Ct≤43, it is positive for A2064G mutation;

3.

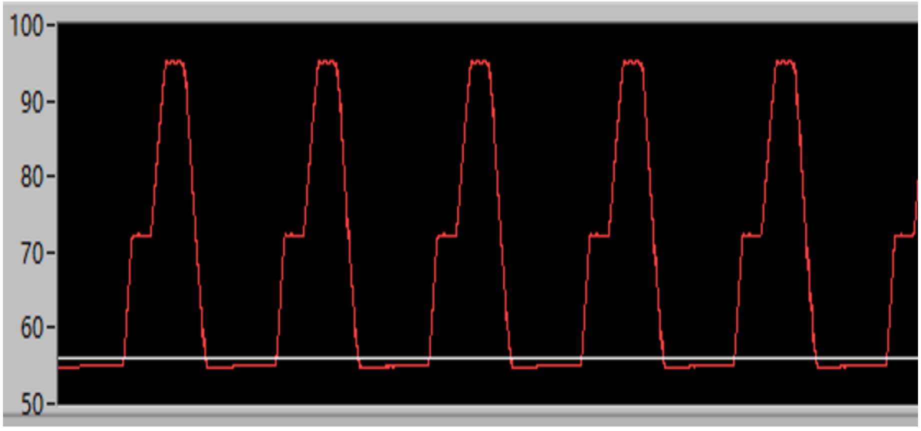

Figure S1. (A)Temperature curve of the homemade instrument. The white line is the temperature condition of fluorescence detection. Whenever the temperature curve drops below the white line, the fluorescence intensity is measured once.

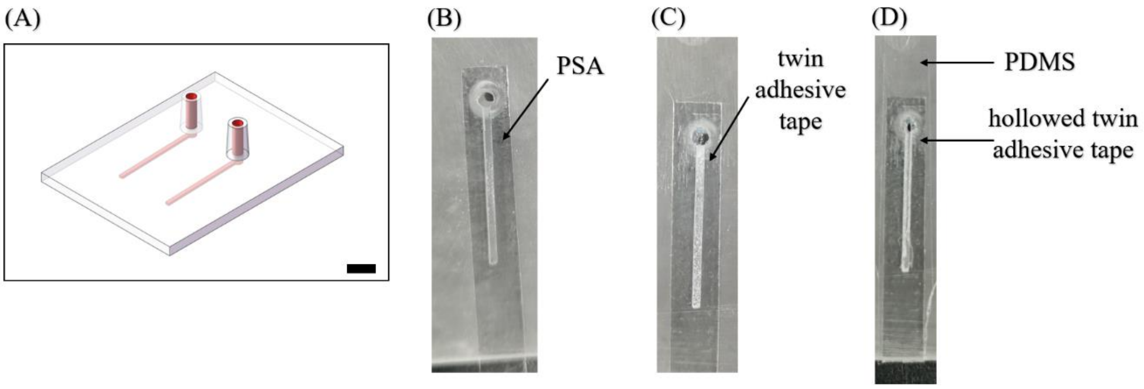

Figure S2. (A) The 3D model of the test chip. scale bar is 10mm. Closed channel by PSA(B), twin adhesive tape(C) and PDMS&hollowed twin adhesive tape(D).

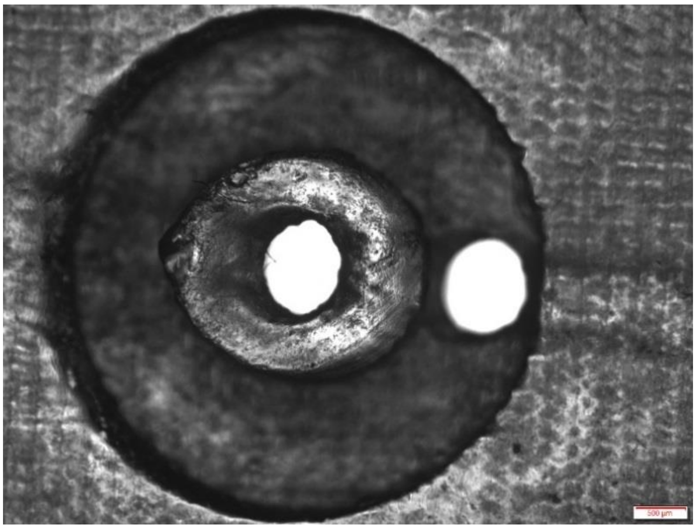

Figure S3. Snapshot of microvalve. scale bar is 500μm.

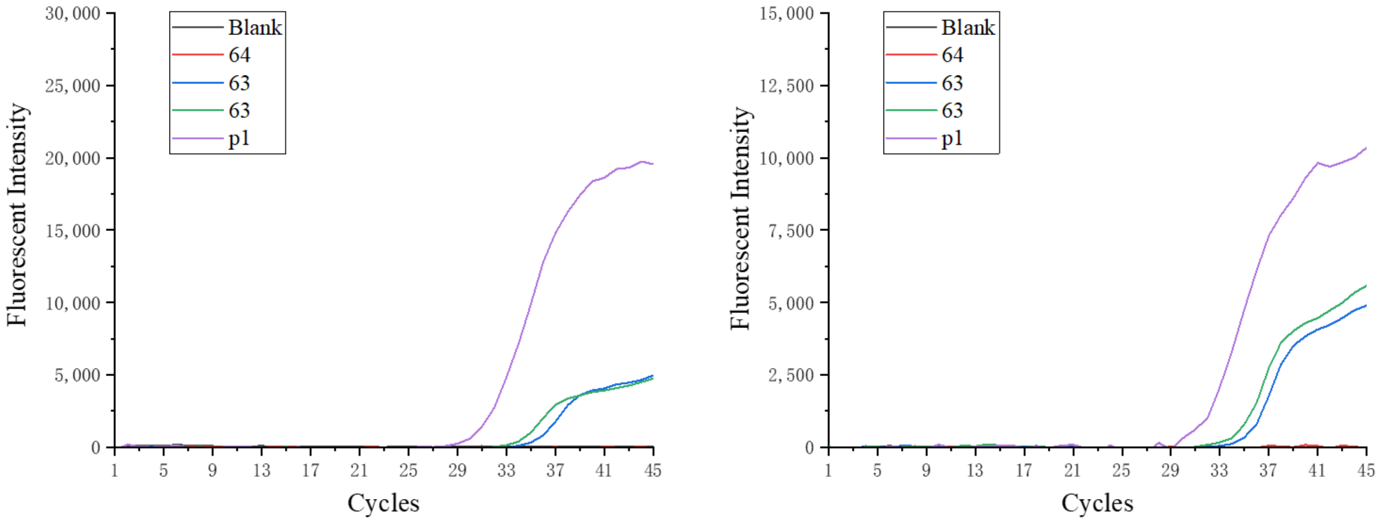

Figure S4. The qPCR curves of other two plasmids samples.

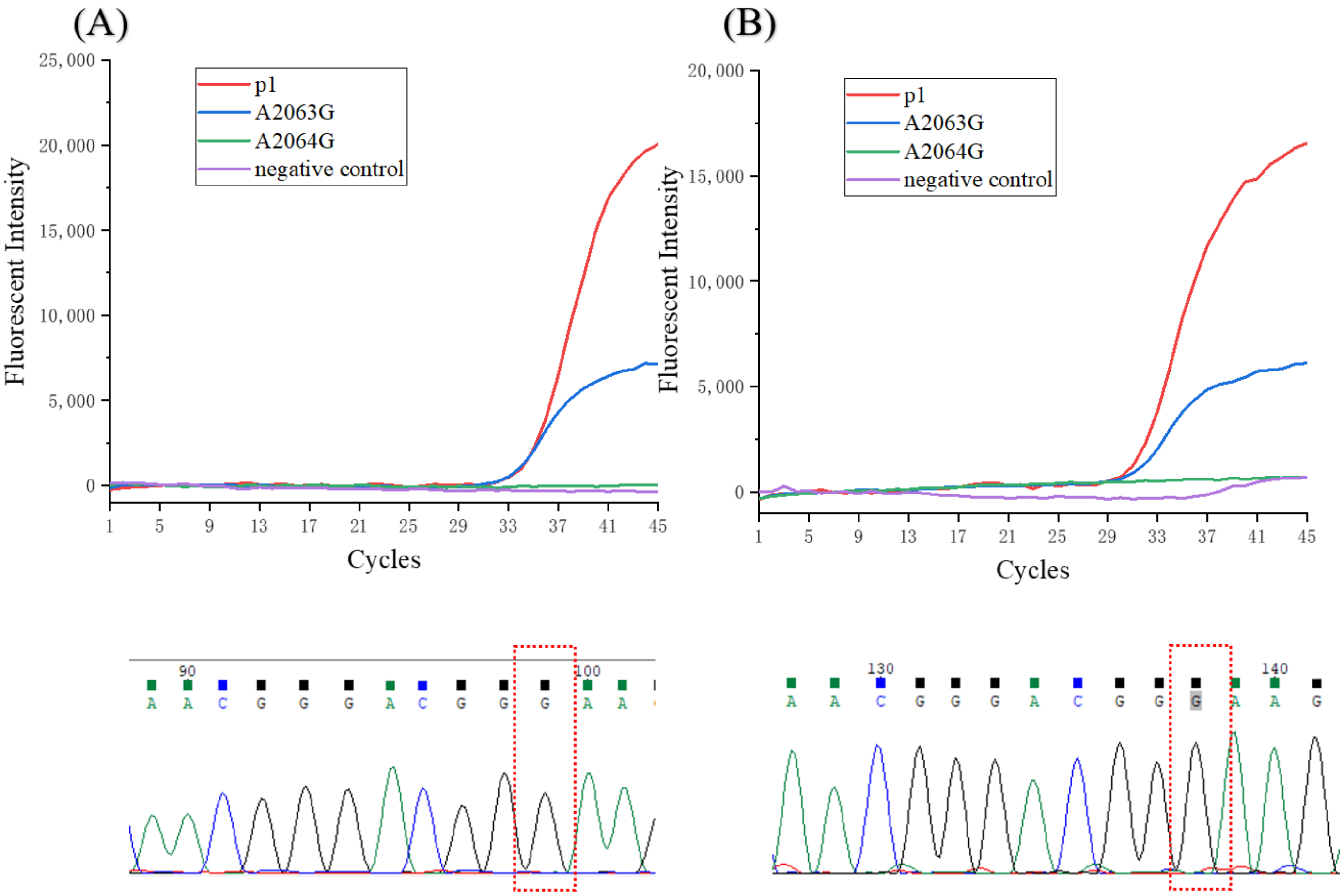

Figure S5. The qPCR curves and sequencing results of other two clinical samples(No.129(A) and No.112(B)).
